# Protection of Sacubitril/Valsartan against Pathological Cardiac Remodeling by Inhibiting the NLRP3 Inflammasome after Relief of Pressure Overload in Mice

**DOI:** 10.1007/s10557-020-06995-x

**Published:** 2020-05-23

**Authors:** Xueling Li, Qin Zhu, Qingcheng Wang, Qinggang Zhang, Yaru Zheng, Lihong Wang, Qinyang Jin

**Affiliations:** 1 Department of Cardiology, Zhejiang provincial People’s Hospital, People’s Hospital of Hangzhou Medical College, Hangzhou, 310014 China; 2 Department of Nephrology, Hangzhou Hospital of Traditional Chinese Medicine, Hangzhou, China; 3 Department of Cardiology, Hangzhou Yuhang Hospital of Traditional Chinese Medicine, Hangzhou, China

**Keywords:** Sacubitril/valsartan, Cardiac remodeling, NLRP3 inflammasome, Pressure unloading

## Abstract

**Background/aims:**

The persistent existence of pathological cardiac remodeling, resulting from aortic stenosis, is related to poor clinical prognosis after successful transcatheter aortic valve replacement (TAVR). Sacubitril/valsartan (Sac/Val), comprising an angiotensin receptor blocker and a neprilysin inhibitor, has been demonstrated to have a beneficial effect against pathological cardiac remodeling, including cardiac fibrosis and inflammation in heart failure. The aim of this study was to determine whether Sac/Val exerts a cardioprotective effect after pressure unloading in mice.

**Methods and results:**

Male C57BL/6 J mice were subjected to debanding (DB) surgery after 8 weeks (wk) of aortic banding (AB). Cardiac function was assessed by echocardiography, which indicated a protective effect of Sac/Val after DB. After treatment with Sac/Val post DB, decreased heart weight and myocardial cell size were observed in mouse hearts. In addition, histological analysis, immunofluorescence, and western blot results showed that Sac/Val attenuated cardiac fibrosis and inflammation after DB. Finally, our data indicated that Sac/Val treatment could significantly suppress NF-κB signaling and NLRP3 inflammasome activation in mice after relief of pressure overload.

**Conclusion:**

Sac/Val exerted its beneficial effects to prevent maladaptive cardiac fibrosis and dysfunction in mice following pressure unloading, which was at least partly due to the inhibition of NLRP3 inflammasome activation.

**Electronic supplementary material:**

The online version of this article (10.1007/s10557-020-06995-x) contains supplementary material, which is available to authorized users.

## Introduction

Aortic stenosis (AS) is the most common type of heart valve lesion in the expanding aged population and can induce left ventricular remodeling due to chronic pressure overload, resulting in the development of heart failure [1]. Emerging clinical trials have established transcatheter aortic valve replacement (TAVR), which exhibits lower invasiveness, risk, and all-cause mortality rate, as the main interventional treatment for AS [2, 3]. However, left ventricular reverse remodeling following successful TAVR is incomplete and is characterized by persistent cardiac fibrosis, inflammation, and hypertrophy, leading to a poor prognosis [4]. Therefore, better strategies for promoting the recovery of early cardiac function post-TAVR are essential to delay heart failure and improve clinical outcomes.

Sacubitril/valsartan (Sac/Val), an angiotensin receptor-neprilysin inhibitor (ARNI) composed of valsartan and sacubitril, has been indicated to be superior to angiotensin-converting–enzyme inhibitors (ACEI), such as enalapril, and to significantly reduce the risk of death and hospitalization in heart failure patients [5, 6]. Recent clinical and animal studies have shown that Sac/Val treatment might be effective in ameliorating cardiac maladaptive remodeling and fibrosis following heart failure [7, 8]. However, the basic mechanism of Sac/Val effectiveness is poorly understood, and little is known about its protective effects against cardiac remodeling after successful TAVR.

Previous studies have revealed that the nucleotide-binding oligomerization domain (NOD)-like receptor protein 3 (NLRP3) inflammasome plays a role as an essential mediator in cardiac remodeling by inducing inflammation and activating the profibrotic pathway [9, 10]. Inhibition of the NLRP3 inflammasome has been implicated as a potential target to prevent pathological cardiac remodeling, including cardiac inflammation and fibrosis, under cardiac injury conditions. However, it is not clear whether Sac/Val exerts a cardioprotective effect post-TAVR by alleviating NLRP3 inflammasome activation.

In this study, we established a mouse model through pressure loading and subsequent unloading to closely mimic TAVR surgery. Our findings confirmed the protective effect of Sac/Val against cardiac hypertrophy, fibrosis, and inflammation after pressure unloading and revealed the underlying mechanisms based on the NLRP3 inflammasome, indicating that Sac/Val might be clinically useful for improving cardiac function and prognosis after successful TAVR surgery.

## Materials and Methods

### Experimental Animals

Six- to eight-week (wk)-old male C57BL/6 J (wild-type) mice were obtained from the Zhejiang Academy of Medical Sciences (Zhejiang, China). All animal experimental protocols were approved by the Institutional Ethics Committee of Zhejiang Provincial Hospital. All procedures were carried out according to the guide for the Care and US National Institute of Health (NIH Publication No. 85–23, revised 1996). All mice were randomly assigned to the Sham (*n* = 10) or aortic banding (AB) group (*n* = 45). At 8 wk post AB surgery, the animal models of pressure overload were proven to be successfully established by echocardiography and histological analysis (Supplemental Fig. 1a–d). Of the 40 surviving mice that underwent AB, 36 were randomly divided into three groups as follows: AB without debanding (DB) surgery, DB + PBS, and DB + Sac/Val. Four mice, including 2 from the sham group and 2 from the 8wk-AB surgery group, were used for Masson staining. A target dose of 24/26 mg Sac/Val was obtained from Novartis (Basel, Switzerland) and dissolved in PBS. To explore the effects of Sac/Val on cardiac fibrosis following DB, mice were administered 60 mg/kg per day Sac/Val (consisting of 28.8 mg/kg per day of Sac and 31.2 mg/kg per day of Val) or equivalent doses of PBS each day by gavage for another 4 wk in accordance with a previous study [7]. The dose of Sac/Val (60 mg/kg/day) was chosen based on published studies [7, 11, 12]. In total, 41 mice, including Sham (*n* = 8), AB (*n* = 10), DB + PBS (*n* = 11) and DB + Sac/Val (*n* = 12), were involved in the result analysis at 12 wk.

### AB and DB Surgery

AB and DB surgeries were performed as previously described [13–15]. In brief, mice were anesthetized with ketamine hydrochloride (50 mg/kg) and diazepam (2.5 mg/kg). The thoracic cavity was opened from the left second intercostal space. A 7–0 silk ligature was tied around the ascending aorta, which was constricted by tying the ligature firmly against a 26 G blunted needle. Then, the needle was promptly removed to yield a constriction. The ligature in sham-operated mice was tied loosely around the aorta. AB mice at 8 wk were then subjected to DB surgery. Following DB surgery, the ligatures of all mice were cut and removed from the aortic arch. The other mice, including those in the sham- and AB- groups, also underwent a second open thoracotomy surgery as controls.

### Echocardiography Evaluation

A transthoracic two-dimensional analysis was performed with a Vevo2100 High-Resolution Micro-Ultrasound System (Visual Sonics, Toronto, Canada) at 8 and 12 wk in mice as previously described [10, 16]. Left ventricular end-systolic diameter (LVESD), left ventricular end-diastolic diameter (LVEDD), and left ventricular (LV) mass were measured in the parasternal long-axis as well as short-axis view. Measurements were taken from more than three beats and averaged. Left ventricular fractional shortening (FS) and ejection fraction (EF) were calculated according to the following formula [17, 18]: EF (%) = [(LVEDD^2^–LVESD^2^)/LVEDD^2^] × 100,FS (%) = [(LVEDD–LVESD)/LVEDD] × 100. Echocardiographic measurements were obtained and analyzed by two investigators who were blinded to the treatment of the mouse.

### Histological Analysis

Mice body weight (BW) and heart weight (HW) from different groups were measured to calculate the ratios of HW/BW. Heart tissues were fixed in 4% paraformaldehyde embedded in paraffin. Several sections at 4–5 μm intervals were obtained and stained with Masson’s trichrome. Heart sections were blocked with bovine serum albumin and incubated overnight with a collagen type I (Col I) primary antibody (Abcam, UK).

### Immunofluorescence Staining

Paraffin sections were deparaffinized and gradually rehydrated in xylene and serial dilutions of ethanol to 70% ethanol. Subsequently, sections were incubated with α-SMA, CD45, NLRP3, and NF-κB antibodies (Abcam, UK) overnight in the dark. After washing twice, the sections were incubated with Alexa Fluor 488- or cy3-labeled goat anti-rabbit IgG (H + L) (Abcam, UK) at room temperature in the dark for 2 h. DAPI was used for nuclear staining.

### Wheat Germ Agglutinin (WGA) and TUNEL Staining

The size and number of the cardiomyocytes in the LV zone were assessed following staining with WGA coupled with Alexa Fluor ™ 488 conjugate (Invitrogen), exactly as described previously [19]. Images were acquired using a Leica fluorescence microscope (Leica, Germany).

A DeadEnd Fluorometric TUNEL Kit (Roche, USA) was used to evaluate cardiomyocyte apoptosis in mouse hearts according to the manufacturer’s instructions as previously described [16]. The apoptotic ratio was calculated as the ratio of the number of TUNEL-positive cells from at least three random fields to the number of DAPI-stained nuclei cells under a Leica fluorescence microscope (Leica, Germany).

### Western Blotting Analysis

Western blotting was performed as previously described [20]. Total protein samples extracted from the hearts of 3–4 mice per group were separated by SDS-PAGE gels and then transferred to PVDF membranes. Each mouse heart protein sample was analyzed in triplicate. After blocking with 5% non-fat dried milk in TBST for 2 h, the membranes were incubated with primary antibodies against NF-κB (1:1000, Abcam, UK), Col I, MMP-2, TGF-β (1:1000, Abcam, UK), NLRP3, Caspase 1, ASC and interleukin-1β (IL-1β), and β-actin (1:1000, Cell Signaling Technology, Inc.) as a loading control. Subsequently, the appropriate HRP-conjugated secondary antibodies were used. Finally, the signals were detected using an ECL chromogenic substrate, and the relative intensities of the protein bands were quantified with Quantity One software (Bio-Rad, Berkeley, CA).

### Quantitative Real-Time PCR (qRT-PCR)

Total RNA from heart tissues was isolated using TRIzol reagent (Invitrogen, Life Technology, USA). One microgram of total RNA from each group was reverse transcribed into cDNA. qRT-PCR was performed in a StepOne Plus Real-Time PCR system (Applied Biosystems) as previously described [10]. The qRT-PCR data were normalized to the average levels of GAPDH. The relative mRNA expression levels were calculated as the 2 − ΔΔCt value. The sequences of the primers used for qRT-PCR are as follows:

Mouse IL-1β: Forward: 5’-AGCTTCAGGCAGGCAGTATC-3′, Reverse: 5’-TCATCTCGGAGCCTGTAGTG −3′; Mouse TNF-α: Forward: 5′- AACTCCAGGCGGTGCCTATG −3′, Reverse: 5′- TCCAGCTGCTCCTCCACTTG −3′; and Mouse GAPDH: Forward: 5′- GAGAAACCTGCCAAGTATGATGAC-3′, Reverse: 5′- AGAGTGGGAGTTGCTGTTGAAG −3′.

### Statistical Analysis

All data were described as mean ± SD. Differences in data were tested by unpaired, two-tailed Student’s t test for two groups or one-way analysis of variance (ANOVA) with post hoc contrasts by the Brown–Forsythe test for multiple comparisons. Prism 6.0 Software (GraphPad) was used for the statistical analyses, and *P* < 0.05 was considered to indicate statistical significance.

## Results

### Sacubitril/Valsartan Preserved Cardiac Function in a Mouse Model of Pressure Unloading

**Fig. 1:**
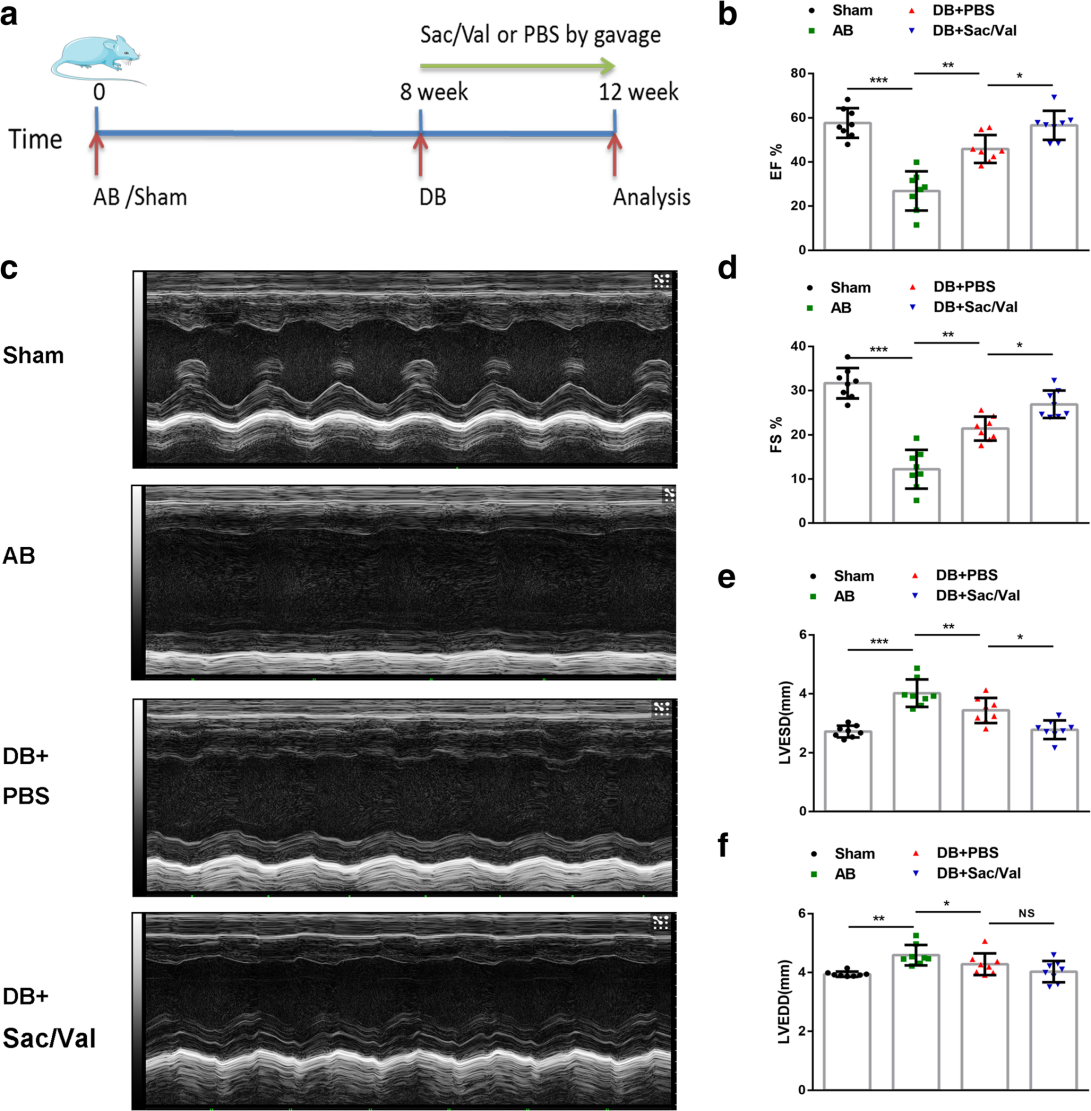
Sacubitril/valsartan preserved cardiac function in a mouse model of pressure unloading. (**a**) Schedule of the 12-wk experiment. The mice underwent DB surgery after 8 wk of AB through tightening and loosening of ligatures around the aorta. Subsequently, all mice were given Sac/Val (60 mg/kg) or equivalent doses of PBS each day by oral gavage for another 4 wk. (**b**) EF, FS (**d**), LVESD (**e**), and LVEDD (**f**) of the mice from the sham, AB, DB + PBS, and DB + Sac/Val groups were measured and calculated at 12 wk (*n* = 8 per group). (**c**) Representative M-mode images of mouse hearts from the four groups are shown. Values are expressed as the mean ± SD. *P < 0.05, **P < 0.01, ***P < 0.001; NS, no significance

To closely mimic AS and therapeutic valve replacement, we established a mouse model with AB for 8 wk and subsequent pressure unloading through DB. Then, the mice were administered with Sac/Val or PBS by oral gavage for 4 wk after DB (for the experimental timeline, see Fig. 1a). Dramatic declines in cardiac function and increases in fibrosis, as evidenced by echocardiography and Masson staining, confirmed the successful establishment of the animal model of pressure overload at 8 wk-long AB (Supplemental Fig. 1 a–d). EF, FS, LVEDD, and LVESD were significantly reduced in 12-wk AB mice compared with 8-wk mice, indicating that cardiac function became progressively worse with longer periods of pressure overload (Supplemental Fig. 1 a, b). Surgical DB after 8-wk AB led to a significant functional improvement as determined by the above parameters following the 4-wk period (Fig. 1b–f). Treatment with Sac/Val after DB significantly altered EF, FS, and LVESD, but not LVEDD (Fig. 1b-f). Overall, these findings suggest that Sac/Val can improve early cardiac function after pressure unloading in mice.

### Sacubitril/Valsartan Attenuated Cardiac Hypertrophy after Pressure Unloading

**Fig. 2:**
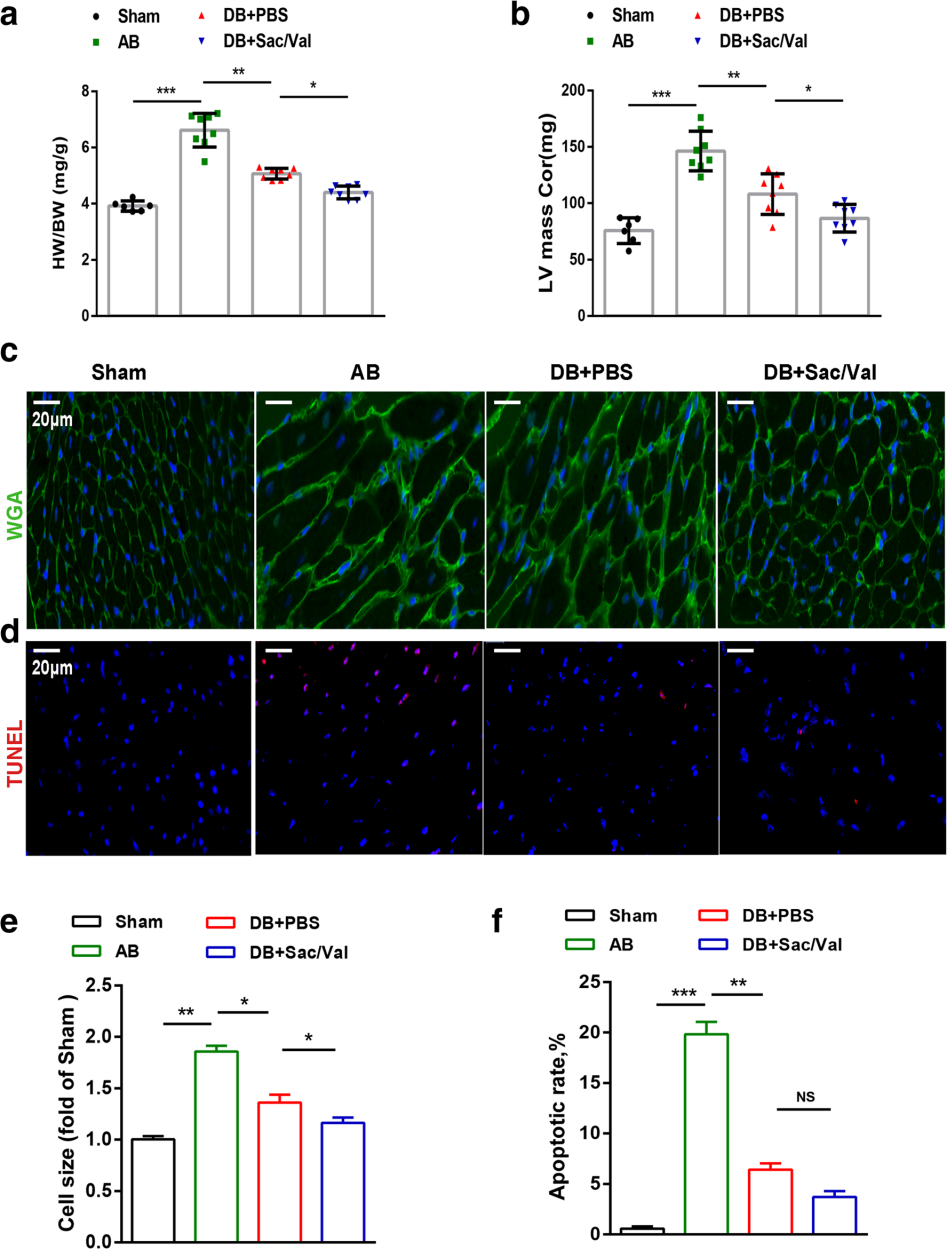
Sacubitril/valsartan attenuated cardiac hypertrophy after pressure unloading. (**a**) Heart weight/body weight (HW/BW) ratios and (**b**) LV mass of the mice from the sham, AB, DB + PBS, and DB + Sac/Val groups were measured and analyzed at 12 wk (n = 8 per group). (**c**) Representative images of WGA staining (green) and DAPI (blue) and quantification of myocyte size (**e**) are shown for mouse hearts from the four groups (*n* = 3 per group). Scale bar =20 μm. (**d**) Representative images of TUNEL staining (red) and calculation (**f**) of the apoptotic index (the ratio of TUNEL-positive nuclei to the total number of cells) are shown for cardiac sections from the four groups at 12 wk (n = 3 per group). Scale bar =20 μm. Mean ± SD. *P < 0.05, **P < 0.01, ***P < 0.001; NS, no significance

To investigate the effect of Sac/Val on the cardiac hypertrophy response to pressure unloading, we also measured the relative heart weight of mice as determined by the ratio of heart weight to body weight (HW/BW). AB was associated with a higher HW/BW than the sham surgery, but the ratio significantly decreased following DB. The HW/BW ratio was significantly lower in the DB + Sac/Val group than in the DB + PBS group (Fig. 2a). This change was confirmed by echocardiographic measurement of the LV mass: the Sac/Val group was associated with markedly lower LV mass than PBS treatment following DB, which itself provided a significant antihypertrophic protection relative to the AB alone administration (Fig. 2b). Moreover, AB significantly increased the cardiomyocyte cross-sectional area, as assessed by WGA staining; however, WGA staining revealed a striking recovery of cardiomyocyte size in the presence of Sac/Val under DB (Fig. 2c and e). The proportion of TUNEL-positive cells in AB-treated mouse hearts was notably higher than that in control hearts and was decreased following DB surgery. Interestingly, a declining trend without a significant difference in apoptotic rate was observed after Sac/Val treatment post DB (Fig. 2d and f).

### Sacubitril/Valsartan Ameliorated Cardiac Fibrosis after Pressure Unloading

**Fig. 3:**
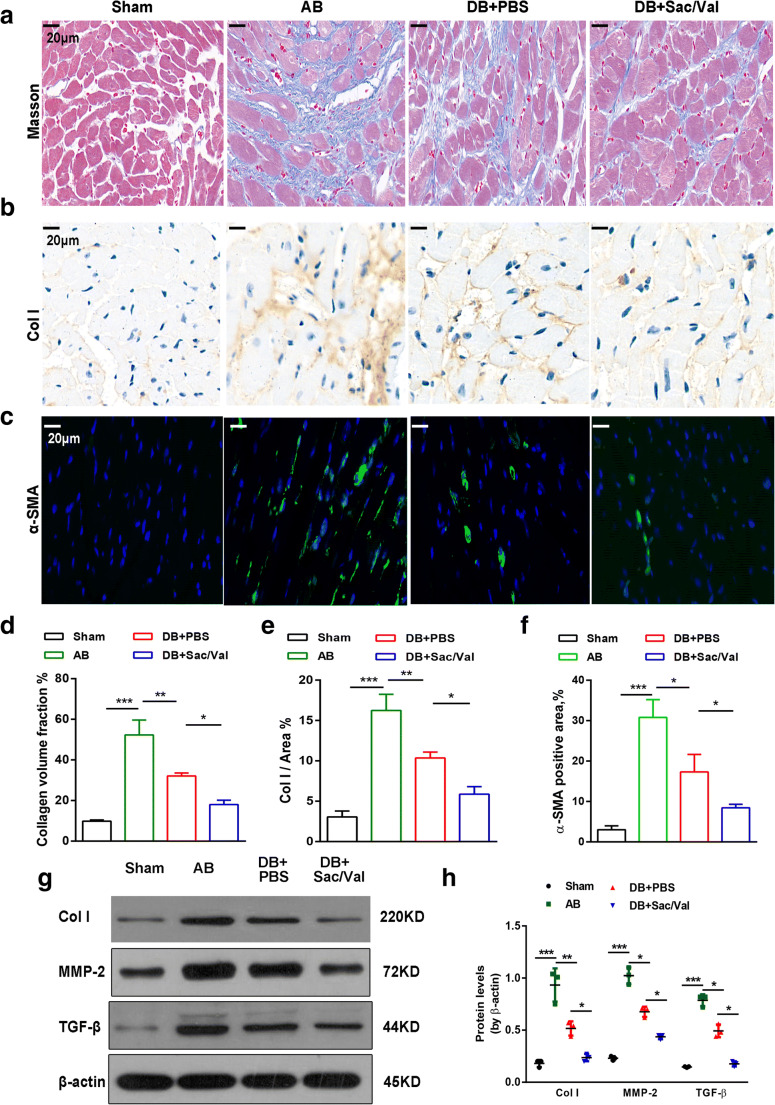
Sacubitril/valsartan ameliorated cardiac fibrosis after pressure unloading. (**a**) Representative images of Masson’s staining, immunohistochemical staining for collagen type I (Col I) (**b**) and immunofluorescence staining (**c**) for α-SMA (green) and DAPI (blue) in mouse hearts from the sham, AB, DB + PBS, and DB + Sac/Val groups are shown. Scale bar =20 μm. (**d**) Quantification of the percentage of collagen volume fraction, Col I area (**e**) and α-SMA-positive cells (**f**) were analyzed and shown (*n* = 3 per group). (**g**-**h**) Western blotting showed the protein expression levels of Col I, MMP-2, and TGF-β and quantified and normalized them to the levels of β-actin (n = 3 per group). Mean ± SD. *P < 0.05, **P < 0.01, ***P < 0.001

Animals subjected to AB surgery showed poor cardiac function and apparent cardiac fibrosis [7]. In accordance with previous research, Masson and Col I staining of mouse hearts showed that AB treatment resulted in obvious cardiac fibrosis and collagen deposition (Fig. 3a, b and d, e). Following induction of DB, these alterations were inhibited, and there was a pronounced reduction in cardiac fibrosis under coadministration with Sac/Val (Fig. 3a, b and d, e). A common marker of cardiac fibroblasts, α-SMA, was used to assess cardiac fibrosis. The proportion of α-SMA-positive cells in DB-treated mouse hearts was significantly lower than that in AB-treated hearts and was further decreased in the presence of Sac/Val (Fig. 3c and f). Additionally, the levels of profibrotic factors, including TGF-β, Col I, and MMP-2, were markedly increased in AB-treated animals compared with untreated animals. In agreement with previous findings [14], treatment with DB surgery was also able to significantly downregulate the levels of these profibrotic factors in mouse hearts. These factors were further decreased by Sac/Val after DB, indicating a protection of Sac/Val against cardiac fibrosis (Fig. 3g and h).

### Sacubitril/Valsartan Impaired the Cardiac Inflammatory Process after Pressure Unloading

**Fig. 4:**
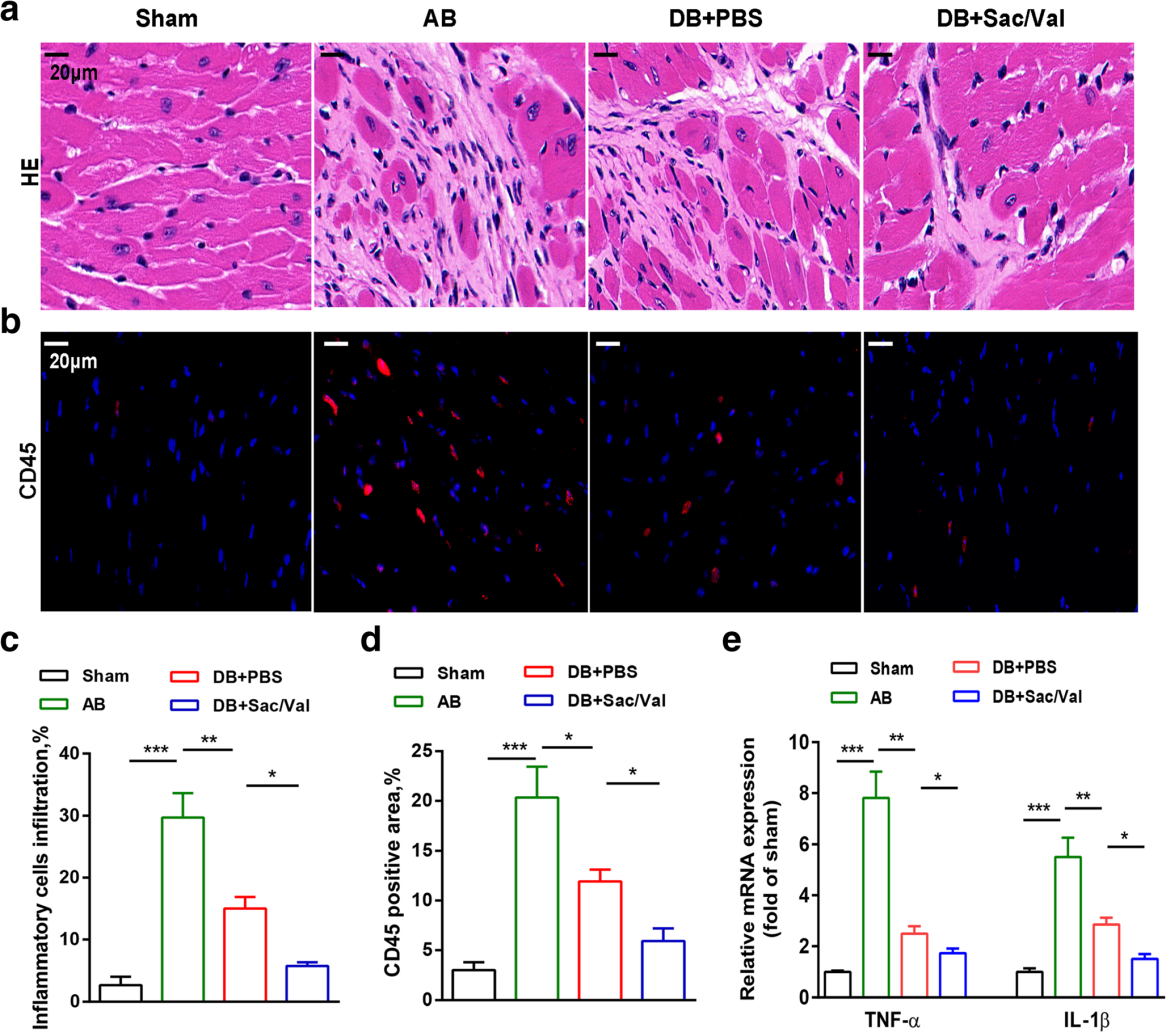
Sacubitril/valsartan impaired the cardiac inflammatory process after pressure unloading. (**a**) Representative images of hematoxylin and eosin (H&E) staining and immunofluorescence staining (**b**) for CD45 (red) and DAPI (blue) in mouse hearts from the sham, AB, DB + PBS, and DB + Sac/Val groups are shown. Scale bar =20 μm. (**c**) Quantification of the percentage of inflammatory infiltration and CD45-positive cells (**d**) were analyzed and presented for mouse cardiac sections among the different groups. (**e**) qRT-PCR analysis of TNF-α and IL-1β mRNA expression levels in heart tissues is shown. (n = 3 per group). Mean ± SD. *P < 0.05, **P < 0.01, ***P < 0.001

Inflammation has been described as a key part of the development of cardiovascular disease. To determine the effect of Sac/Val on the inflammatory response, we analyzed the infiltration of inflammatory cells and the levels of inflammatory cytokines. Hearts from AB-treated mice exhibited increased inflammatory infiltration as determined by hematoxylin-eosin staining; however, this infiltration was blunted in mouse hearts following DB surgery, especially in Sac/Val-treated mouse hearts (Fig. 4a and c). These alterations were confirmed by CD45 staining, which showed marked increases in the total number of CD45+ cells in hearts from AB-treated mice versus those from sham mice. Furthermore, there were significantly fewer CD45+ heart inflammatory cells in DB + Sac/Val mice than in DB + PBS mice (Fig. 4b and d). The mRNA expression levels of proinflammatory cytokines, including IL-1β and TNF-α, were dramatically decreased in Sac/Val-treated mice that underwent DB surgery (Fig. 4e). These results indicate that Sac/Val can alleviate inflammatory cell infiltration and inhibit the expression of inflammatory cytokines.

### Sacubitril/valsartan Inhibited NLRP3 Inflammasome Activation after Pressure Unloading

**Fig. 5:**
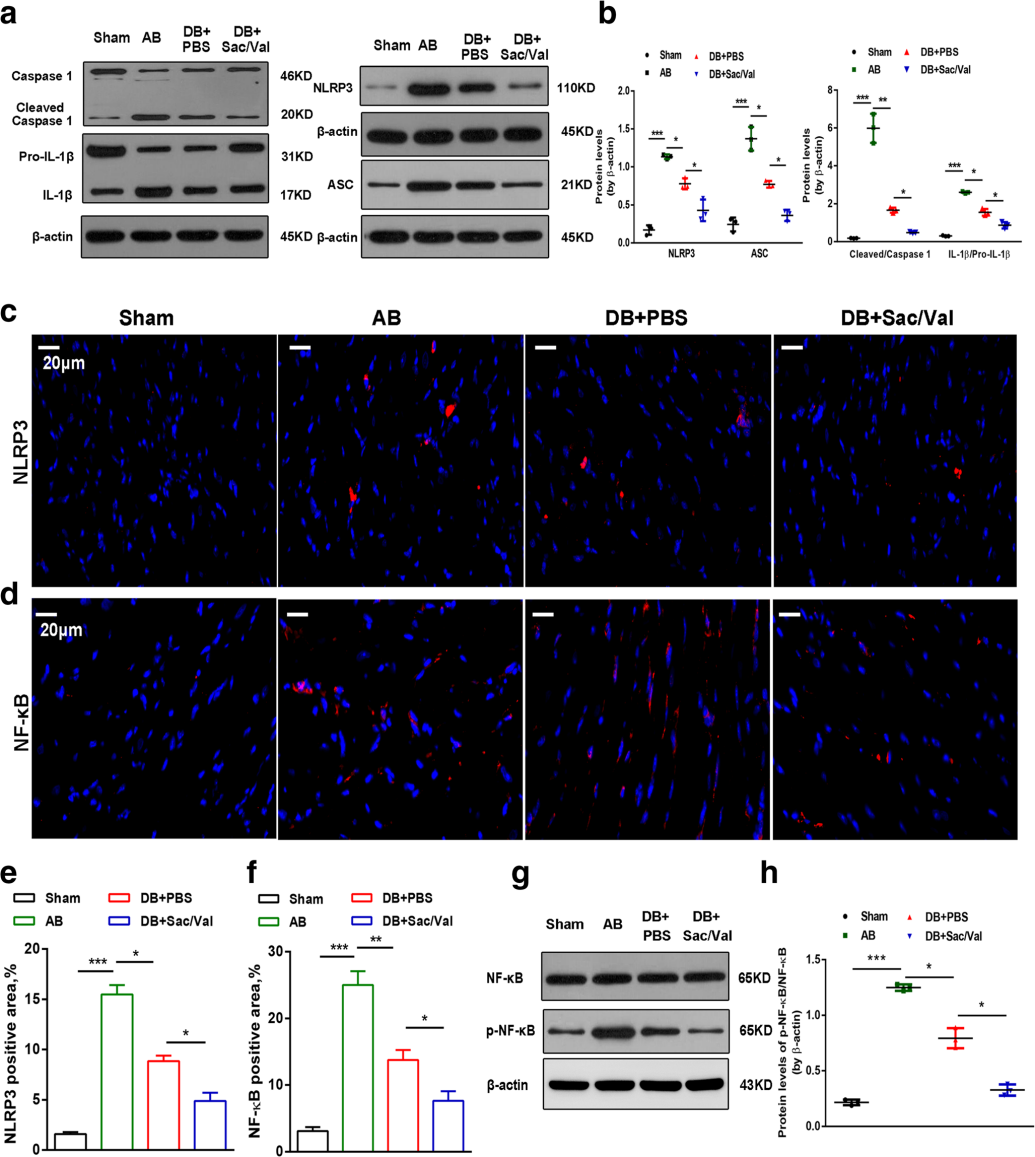
Sacubitril/valsartan inhibited NLRP3 inflammasome activation after pressure unloading. (**a**) Western blotting shows the protein expression levels of NLRP3, ASC, pro-IL-1β, IL-1β, caspase-1, and cleaved caspase-1 from the sham, AB, DB + PBS, and DB + Sac/Val mouse hearts. (**b**) The protein levels of NLRP3, ASC, and cleaved/caspase-1 and the ratio of IL-1β to pro-IL-1β were quantified and normalized to the levels of β-actin. (**c**) Immunofluorescence images for NLRP3 (red) and (**d**) NF-κB (red) as well as DAPI (blue) in mouse hearts from the 4 groups are shown. Scale bar =20 μm. (**e**, **f**) Quantification of the percentage of NLRP3- and NF-κB-positive cells was analyzed and presented in mouse cardiac sections among different groups. (**g**, **h**) NF-κB and p-NF-κB levels in heart tissues are shown by Western blotting, and the ratio of p-NF-κB to NF-κB is quantified and normalized to the levels of β-actin. (n = 3 per group). Mean ± SD. *P < 0.05, **P < 0.01, ***P < 0.001

Considerable evidence has demonstrated that activation of the NLRP3 inflammasome contributes to cardiac fibrosis [10, 21]. To explore whether Sac/Val could suppress the activation of the NLRP3 inflammasome, including NLRP3, ASC, caspase 1, and IL-1β after pressure unloading, we detected these protein levels in mouse hearts among the different groups using Western blotting. The levels of NLRP3 and ASC were significantly decreased in DB + PBS-treated groups compared with those in the AB-treated groups, which were lower in that co-treatment with Sac/Val (Fig. 5a and b). The ratios of IL-1β to pro-IL-1β and cleaved caspase 1 to caspase 1 were also lower in the DB + Sac/Val-treated mice than either the AB- and DB + PBS-treated animals(Fig. 5a and b). Moreover, we also detected the activation of NLRP3 in mouse hearts by immunofluorescence. Our results revealed that elevated NLRP3 levels were observed in AB-treated hearts and that a marked decline was shown in DB + Sac/Val-treated hearts compared with DB + PBS-treated hearts (Fig. 5c and e). NF-κB has been shown to be an upper molecule of the NLRP3 inflammasome involved in inflammation and fibrosis. AB promoted the nuclear translocation of the NF-κB protein in mouse hearts, which was suppressed by DB, especially under Sac/Val administration (Fig. 5d and f). Similarly, Sac/Val treatment alleviated the ratio of p-NF-κB to NF-κB in DB-treated mouse hearts, as assessed by Western blotting (Fig. 5g and h). Accordingly, the above data indicate that Sac/Val might be capable of inhibiting NF-κB-mediated NLRP3 inflammasome activation after pressure unloading in mouse hearts.

## Discussion

Ongoing studies have definitively identified TAVR as the dominant interventional treatment for AS [2, 3]. However, several trials indicated that myocardial dysfunction-related processes, including cardiac fibrosis and hypertrophy, are persistent after stenosis relief in patients with severe AS, leading to a poor prognosis [22]. At present, medications against myocardial hypertrophy and fibrosis have not been recommended after TAVR, possibly due to a lack of sufficient data or to declines in renal function. Mounting evidence has suggested that the beneficial impact of Sac/Val, the ARNI over standard of care (the ACEI or ARB) exerted an improvement in the morbidity and mortality of heart failure patients, as recommended by the current American and European guidelines [8, 23]. Whether ARNI can help to improve effective cardiac remodeling post TAVR and the potential basic mechanisms are currently under investigation.

This study intended to dissect the effects of Sac/Val after relief of pressure overload in mice. We found that Sac/Val, as expected, significantly improved protection against cardiac functional decline in a mouse model of pressure unloading. Cardiac fibrosis and inflammation were impaired in DB-treated mouse hearts compared with AB-treated hearts, and these changes were further suppressed in the presence of Sac/Val treatment. In addition, we found that Sac/Val protected mice against cardiac fibrosis and inflammation following DB surgery by alleviating NF-κB-mediated NLRP3 inflammasome activation.

The extent of cardiac functional recovery is dependent on the duration of pressure overload [24]. Early DB in mice subjected to 6 wk of AB results in a rapid and complete recovery of cardiac structure and function with reduced fibrosis and hypertrophy [25]. However, regression after long-lasting pressure overload evoked by removal of the aortic ligature after at least 8 wk has been found to lead to incomplete restoration of ventricular function and persistent structural changes during 4- or 6-wk-long follow-up periods [26]. We also observed a worse cardiac function in the 12-wk AB group than that in the 8-wk AB group, suggesting slower and incomplete regression after DB. To better explore the effect of Sac/Val on cardiac remodeling after pressure unloading, we generated a mouse model with AB for 8 wk and DB for 4 wk based on previous findings [14, 24]. Undoubtedly, our data confirmed that DB resulted in significant recovery of ventricular function and amelioration of structural decline associated with cardiac fibrosis, hypertrophy and inflammation, and mice concomitantly treated with Sac/Val exhibited better and earlier cardiac functional recovery than those treated with PBS.

Some preclinical and clinical studies have shown that Sac/Val is safer and more efficient than the ACEI enalapril for improving primary outcomes and lowering mortality rates in heart failure [8, 27]. A recent clinical report has shown that ACEI treatment post TAVR offers a global protective effect and decreases the rate of cardiac mortality; these effects are at least partly related to improving cardiac remodeling [28]. However, the effect of Sac/Val on clinical outcomes following successful TAVR remains unclear. We found that Sac/Val further improved cardiac functional parameters, excluding LVEDD, after relief of pressure overload in mice, most likely due to the short-term effects and relatively small number of animals. Sac/Val therapy may be a viable clinical medical strategy for post-TAVR patients because of its beneficial effects on cardiac function.

Several reports have indicated that the benefits of Sac/Val are associated with attenuation of cardiac hypertrophy and fibrosis [29, 30]. In agreement with these findings, Sac/Val also suppressed cardiac hypertrophy, thus exhibiting an antihypertrophic effect, in this study, reducing heart relative weight, LV mass, and myocardial cell size after pressure unloading in mice. Interestingly, Sac/Val therapy post DB tended to decrease apoptosis, as assessed by TUNEL staining, but the decrease was not significant. The relatively short duration of AB may have led to apoptosis of relatively few cells after DB surgery and thus may have influenced the antiapoptotic effect of Sac/Val. Cardiac fibrosis is known to play a vital role in LV pressure loading [7]. Consistent with this role, we found that profibrotic factor levels, collagen production, and cardiac fibroblast activation associated with cardiac fibrosis apparently decreased in response to Sac/Val treatment post DB. Collectively, these findings clarify that treatment with Sac/Val has a protective effect against cardiac hypertrophy and fibrosis following DB in mice.

A previous report has illustrated that Sac/Val more strongly suppresses proinflammatory cytokine expression and inflammatory cell infiltration than ACEI, contributing to improved cardiac outcomes [31]. Here, we observed that infiltration of heart inflammatory cells was obviously ameliorated by Sac/Val treatment after DB surgery. Consistent with previous findings, the mRNA levels of proinflammatory cytokines were dramatically decreased in the DB + Sac/Val group compared with the DB + PBS group. The NLRP3 inflammasome, an intracellular multiprotein complex, including NLRP3, caspase-1, IL-1β and ASC, has been implicated as a principal mediator of pathological ventricular remodeling, referring to inflammation and fibrosis [10, 21]. Briefly, under cardiac injury conditions, NLRP3 forms a complex with ASC, which triggers the production of cleaved caspase-1 and IL-1β, leading to cardiac inflammation and the activation of TGF-β-mediated cardiac fibrosis. With regard to this pathway, we found that Sac/Val reduced the expression levels of NLRP3 and ASC and inhibited the activation of caspase-1 and IL-1β after relief of pressure overload in mouse hearts. The NF-κB pathway is a priming signal for activation of the NLRP3 inflammasome [32–34]. Furthermore, our experiments have shown that Sac/Val inhibits the expression and activation of NF-κB [35]. Although the molecular mechanism by which Sac/Val inhibits cardiac fibrosis and inflammation following pressure unloading is poorly understood, we believe that Sac/Val protects against cardiac remodeling at least in part by suppressing NF-κB-mediated NLRP3 inflammasome activation.

Some limitations of this study should be noted. The working mechanism of Sac/Val in regulating NF-κB/NLRP3 signaling was investigated only in vivo; thus, future studies are needed to validate these targeted cells and specific molecular mechanisms in vitro. Moreover, since the beneficial effect of Sac/Val on mouse heart remodeling post-pressure unloading has been stated, the optimal timing and dose of Sac/Val need to be resolved in further studies. There is also need to observe the dynamic changes in NF-κB/NLRP3 signaling molecule levels at different time points in animals and clinical studies.

The data from this study suggest that Sac/Val can prevent cardiac remodeling, as indicated by improvements in cardiac function and decreases in cardiac fibrosis, hypertrophy, and inflammation. We found that the protective effect of Sac/Val was related to modulation of NF-κB-dependent inhibition of NLRP3 inflammasome activation after pressure unloading in mice. To the best of our knowledge, this is the first study to show that early treatment with Sac/Val following relief of AB is likely to be an important clinical therapy for use after TAVR.

## Electronic Supplementary Material

ESM 1 (DOCX 28774 kb)

## Data Availability

The data will be made available by the corresponding author upon request.
