# Supplementary material for: Protection of Sacubitril/Valsartan against Pathological Cardiac Remodeling by Inhibiting the NLRP3 Inflammasome after Relief of Pressure Overload in Mice
Source: Cardiovasc Drugs Ther. 2020 May 23;34(5):629–40. doi: 10.1007/s10557-020-06995-x (PMC7497317; doi:10.1007/s10557-020-06995-x)
Supplement: Supplementary file 1 — (DOCX 28774 kb) [file 10557_2020_6995_MOESM1_ESM.docx]

**Supplementary Material**

**Supplemental Figure 1.The animal model of pressure load was successfully established at 8 weeks. (A-B)**Cardiac function including EF, FS, LVESD, LVEDD from 8wk and 12wk AB group were measured and calculated, indicating the success of the animal model of pressure load and more worse function at 12 week (n=8 in 0 and 12wk groups; n=14 in 8wk group).**(C-D)** Representative images of Masson’s staining and quantification of the percentage of collagen volume fraction were shown at 8week following AB(n=3 per group). Mean ± SD. * P < 0.05, ** P<0.01, *** P<0.001.
